# HIV treatment cascade among female sex workers in Ethiopia: Assessment against the UNAIDS 90-90-90 targets

**DOI:** 10.1371/journal.pone.0294991

**Published:** 2023-12-13

**Authors:** Saro Abdella, Meaza Demissie, Alemayehu Worku, Merga Dheresa, Yemane Berhane

**Affiliations:** 1 HIV and TB Research Directorate, Ethiopian Public Health Institute, Addis Ababa, Ethiopia; 2 Department of Global Health and Health Policy, Addis Continental Institute of Public Health, Addis Ababa, Ethiopia; 3 School of Public Health, College of Health Sciences, Addis Ababa University, Addis Ababa, Ethiopia; 4 School of Nursing and Midwifery, College of Health Sciences, Haramaya University, Harar, Ethiopia; 5 Department of Epidemiology and Biostatistics, Addis Continental Institute of Public Health, Addis Ababa, Ethiopia; University of Zimbabwe Faculty of Medicine: University of Zimbabwe College of Health Sciences, ZIMBABWE

## Abstract

**Background:**

HIV treatment cascades for HIV-positive female sex workers (FSWs) have been challenged by the overlapping stigma and discrimination associated with both their sex work and HIV status. This study aims to assess the proportion of HIV-positive FSWs who access care and treatment in Ethiopia.

**Method:**

A cross-sectional study with a respondent-driven sampling technique was used to enroll 6,085 female sex workers from January to June 2020. Interviews were conducted to assess the FSWs’ HIV status awareness and access to ART. A blood sample was drawn to determine the current HIV status and viral load level. Logistic regression was run to identify factors associated with FSWs’ HIV status awareness.

**Results:**

Of the total 1140 HIV-positive FSWs, 50.38% knew they were HIV positive; 92.88% of those who knew their status were on ART, and 91.68% of those on ART had attained viral suppression of less than 1000 copies per milliliter. The adjusted odds of knowing HIV status was 3.20 (95% CI; 2.00, 5.13) among those aged 35 years and older, 1.81 (95% CI; 1.05, 3.12) among widowed, and 1.73 (95% CI; 1.28, 2.32) in those who did not perceive the risk of HIV acquisition.

**Conclusion:**

Only about half of HIV-positive FSWs knew they were HIV positive. More than 90% of those who knew their status were put on ART and achieved viral suppression. The weakest point in achieving HIV control among FSWs is the identification of those living with HIV.

## Introduction

The prevalence of HIV among female sex workers (FSWs) could be as high as 70%, with a considerable variation across countries and regions [1]. The prevalence among FSWs in the Horn of Africa is considerably high; for example, HIV prevalence in FSWs was 17.3% in South Sudan [1], 18.7% in Ethiopia [2], and 33% in Uganda [3]. Besides, access to HIV prevention services is disproportionally poor among FSWs due to several individual and structural factors, making them 26 times more likely to contract HIV than women in the general population [4].

Cognizant of the higher HIV risk and vulnerability in FSWs, the Joint United Nations Program on HIV/AIDS (UNAIDS) provided specific guidance on HIV prevention in the context of sex work. The guidance reinforces three interdependent pillars: advocating access to HIV prevention, treatment, and care; creating supportive environments and partnerships for universal access to services and alternative occupations; and actions to address structural issues related to sex work and HIV [5, 6]. In addition, renewed targets were set for 2020, which include that 90% of all people living with HIV would have known their HIV status; of those who knew their status, 90% should have been on ART; and 90% of all on ART should have attained viral suppression (1000 copies/mili litter) [7]. Even though the baseline performance in the treatment cascade varied across countries, the target did not account for the differences at the starting point [8]. Studies in Africa indicate variable success in the three targets; some show lower access to testing but better coverage in ART and viral suppression [9], while others report higher access to HIV testing but lower coverage of ART initiation and viral suppression [10], There was also a study that illustrated lower coverage in all three [11].

The HIV treatment cascade in HIV-positive FSWs has been significantly challenged by stigma and discrimination due to reasons related to both sex work [12] and HIV status [13]. FSWs prefer not to seek HIV testing services because they fear being identified as FSWs and HIV-positive. In addition, the lack of social support negatively affects access and adherence to ART [12, 13]. The stigma and the discriminatory attitudes and behaviors of the health care providers are also among the key barriers to HIV test uptake [14, 15]. Other commonly reported factors contributing to late HIV testing and delayed treatment initiation among FSWs are lack of transportation, time, and low service quality [16–18].

According to a population-based HIV impact assessment survey conducted in urban Ethiopia from 2017–2018, 79% of people living with HIV were aware of their HIV status, 97.1% were on ART, and 87.6% had achieved viral suppression [19]. A retrospective urban facility-based study done in central Ethiopia in 2021 reported 92% viral suppression among people living with HIV(PLHIV) [20], while a meta-analysis to evaluate HIV treatment success in Ethiopia showed 15.9% HIV treatment failure [21]. Though improved access to HIV prevention measures, including the HIV test and treat programs, is believed to reduce HIV new infections in sex workers [22], no specific study has been conducted to report the HIV treatment cascade of FSWs in the country. Therefore, this study intended to assess FSWs’ access to HIV testing services, ART, and viral suppression status. In addition, the study will also report factors associated with HIV status awareness among FSWs in Ethiopia.

## Materials and methods

### Study design, techniques, and period

A cross-sectional study design with a respondent-driven sampling (RDS) technique to recruit FSWs was used from January to June 2020. RDS is a special snowball sampling technique used to recruit socially hidden populations that have evolved into a chain-referral sampling method with link-tracing designs [23]. It combines a data collection procedure through chain referral and analysis with a mathematical model that weights the sample to compensate for the fact that the sample was collected in a non-random way [24].

### Study population, setting, and sample size

Women aged 15 years and older who received money or other benefits for sex with four or more people within the last 30 days were eligible to participate in this study. In addition, FSWs’ willingness to participate in the study and refer others in their network for participation were other criteria for enrolment in the study.

Using a single population proportion formula with a confidence interval of 95%, a margin of error of 5%, and a proportion of 50% (no study to assess treatment access among FSWs was conducted previously in Ethiopia), the estimated sample size was 385 per city with a total sample size of 5,775. Sixteen randomly selected cities were included in the study [25].

### Study procedures and data collection

We initiated the study by purposively selecting 98 seeds, 5–12 seeds per city. The initial seeds were selected considering the types of sex workers (venue-based, street-based, phone-based), age categories, places of work, and geographic locations of the sites. These seeds recruited peers to form the first wave, then recruited other peers for the second wave. The process continued through successive waves of recruitment until the target sample size in each city was reached.

The seeds were given orientation about the study; consent was obtained; they were interviewed; blood was drawn; and they were trained to invite friends in their social network to the study. Three coupons were given to each seed to invite their peers. Participants were expected to visit the study sites two weeks after the coupon dispatch date. The seeds were returned after two weeks to receive their incentive for recruiting others, compensation for their transportation, and the result of their blood drawing.

Upon arrival of study participants for an initial visit, the study team checks coupon validity and provides screening questions to assess eligibility to participate in the study. After passing the screening and obtaining written consent, participants were administered face-to-face interviews using a structured questionnaire built-into ODK. The questionnaire was designed in such a way that no data is missed by making it compulsory to answer the previous question before proceeding to the next one. The data collection tool included variables related to demographic, social, behavioral, HIV/STI history, and service uptake. In addition, access to HIV testing service was obtained from a response to a question that assessed if an FSW had ever tested for HIV or not; and ART access was evaluated by the FSW’s self-report of being on ART or not.

After the interview, participants were provided counseling to take an HIV test. Whole blood from a finger-prick was collected and tested for HIV using the national HIV testing algorithm (HIV1/2 stat-pak—assay one, Abone—assay two, and SD bioline—assay three). In addition, venous blood was drawn from all HIV-positive participants and every 10^th^ HIV-negative participant for viral load testing and quality assurance purposes, respectively. The drawn blood was transported to the national reference laboratory found at the Ethiopian Public Health Institute. A senior laboratory professional at the reference laboratory tested the samples collected to assess the test result agreement using the same test algorithm deployed at the site level.

Finally, participants had collected their incentives for participation and transportation. They were also given three coupons and oriented to invite peers in two weeks before their second visit. On the second visit, they were compensated for transportation and each successful recruitment. HIV-positive FSWs received their viral load test results and were escorted to health facilities of their preference to obtain HIV care and treatment.

### Dependent and independent variables

Knowing HIV status, being linked to care, and being on ART were the three major variables used to report the HIV treatment cascade, and all were obtained from the FSW’s self-report.

To calculate the proportion of HIV-positive FSWs who knew their HIV status, the first 90, we took the number of HIV-positive FSWs who were aware of their HIV status before the study and divided it by the total number of HIV-positive FSWs identified during the study period.

The proportion of HIV-positive FSWs on ART among those who knew their HIV status, referred to as the "second 90," was calculated by dividing the total number of HIV-positive FSWs who reported being on ART by the number of HIV-positive FSWs who were aware of their HIV status before our survey.

The third 90, or the proportion of FSWs with HIV viral suppression among those on ART, was computed by dividing the total number of FSWs who reported being on ART and had a viral load of less than 1000 copies/mL by the total number of FSWs who reported being on ART.

A denominator for the proportions of "linkage to care among all HIV positive FSWs,” "on ART among all HIV positive,” and "viral suppression among all HIV-positive FSWs" was the total number of HIV-positive FSWs identified during the survey period.

Knowing HIV status was considered a dependent variable, while age, marital status, average monthly income, educational status, HIV acquisition risk perception, and history of STIs were independent variables for analysis.

HIV acquisition risk perception was measured through the self-reported responses of FSWs to a question that asked whether they thought their work exposed them to HIV infection or not.

### Data analysis

Data were cleaned and analyzed using the Respondent Driven Sampling Analysis Software (RDSAT) version 6.4. Diagnoses for the RDS assumptions were also conducted. The majority of the participants were recruited from seeds that produced two to six waves. The wave length had reached up to 16. HIV status and consistence condom use responses were converged to their respective population estimates at each study site. There was no bottleneck, and low homophily of 0.3 for HIV status and 0.2 for consistent condom use were observed [25].

The data were weighted using the social network of FSWs in RDS II to estimate the proportion of FSWs who were aware of their HIV-positive status, the proportion of FSWs on ART among those who were aware of their HIV-positive status, and the proportion of FSWs who were virally suppressed among those on ART. We used STATA 14.1 to compute the three 90s with a 95% confidence interval. All proportion outputs were computed after the data were weighted using RDS II. Finally, the outputs were exported to an Excel sheet to draw graphs.

Binary logistic regressions were run to determine factors associated with access to HIV testing in FSWs. Chi-square tests were used to compare the independent associations of each variable with HIV test access. Variables that either fulfill the statistical criteria of a p-value of less than 0.25 or those considered important from the programmatic point of view were included in the regression model to compute the adjusted odds ratio (AOR). A collinearity test was conducted, and variables with a Pearson’s correlation coefficient greater than 0.5 were removed from the logistic regression model to ensure that each variable in the analysis represents a unique concept. Only non-overlapping variables that could explain the access to HIV tests were included in a mixed-effect logistic regression model where the difference due to the study sites was controlled. A P-value of less than 0.05 was considered to report a significant association between dependent and independent variables.

### Ethical considerations

Only mentally capable FSWs who could decide and volunteer to participate in the study were enrolled. A written informed consent form that was used to obtain consent from participants included all elements of a standard consent form: the purpose of the study, the risks and benefits, the confidentiality of the data they provide, their rights to withdraw, and contact addresses in case they need them. Mature minors, aged between 15 and 18 years, who were responsible for their livelihood were involved in the study. Codes were used to register participants; no personal identifying information was collected. The study protocol was reviewed and approved by the Ethiopian Public Health Institute’s Review Board (Ref. EPHI 6·13/517).

## Results

We recruited 98 seeds and distributed 15,890 coupons. The average coupon return rate was 38.40%, and 6,085 eligible FSWs were successfully enrolled in the study. Not passing the coupons to peers for enrolment, not being ready to visit the study sites, and missing the study place could be some of the potential reasons for the remaining unreturned coupons. Fifty-two percent of the FSWs were younger than 25 years, and 83.93% of them had sex before the age of 18. Most (58.50%) of the FSWs did not complete primary school, and 37.17% divorced. The average monthly income of 42.74% of FSWs was less than 3000 Ethiopian Birr (ETB) (Table 1).

**Table 1:** Socio-demographic characteristics of FSWs in Ethiopia, 2020 (N = 6085).

Variables	Categories	Number	Percent
**Age in year**	<25	3190	52.42
** **	25–35	2413	39.65
** **	≥ 35	482	7.92
**Age at first sex in year**	≥ 18	978	16.07
** **	< 18	5107	83.93
**Marital status**	Not Married	2944	48.38
** **	Currently married	231	3.80
** **	Separated	372	6.11
** **	Divorced	2262	37.17
** **	Widowed	276	4.54
**Highest grade attended**	Secondary school and above (≥ grade 9)	1471	24.17
** **	Primary school (< grade 9)	3560	58.50
** **	Not able to read and write	1054	17.32
**Average monthly income (ETB)**	More than 3,000	3484	57.26
	Less than 3,000	2601	42.74

### Knowing HIV status

Overall, 89.56% (95% CI; 88.55, 90.53) of the 6,085 FSWs who participated in this study reported being ever tested for HIV at least once in their lifetime. However, among the FSWs who tested positive for HIV (n = 1140) in the current study, only 50.38% (95% CI;46.70, 54.07) knew their being HIV-positive before this study. Also, 33.25% (95% CI; 29.84, 36.84) of current HIV-positive FSWs had received HIV-negative test results during their previous test, and 1.45% (95% CI; 0.90, 2.34) did not receive their test results at all. Some 14.89% (95% CI; 12.28, 17.94) of them had never been tested for HIV before the study time (Table 2).

**Table 2:** HIV test access and knowledge of HIV status among female sex workers in Ethiopia, 2020.

HIV test result (n = 6085)	Self-reported previous HIV test	Number (%, 95% CI)	Test Result, Number (%, 95% CI)		
			Not Received N (%; 95% CI)	Negative N (%; 95% CI)	Positive N (%; 95% CI)
**HIV negative (n = 4945)**	Ever tested	4520 (90.54%, CI: 89.50, 91.58)	42 (1.02%, CI: 0.71–1.46)	4478 (98.99%, CI: 98.54–99.29)	-
	Never tested	425 (9.46%, CI: 8.45, 10.58)	-	-	
**HIV positive (n = 1140)**	Ever tested	992 (85.11, CI: 82.06, 87.72)	24 (1.45%, CI: 0.90–2.34)	393 (33.25%, CI: 29.84–36.84)	575 (50.38%, CI:46.70–54.07)
	Never tested	148 (14.91%, CI: 1230, 17.97)	-	-	-

### HIV status and viral suppression

**Fig 1:**
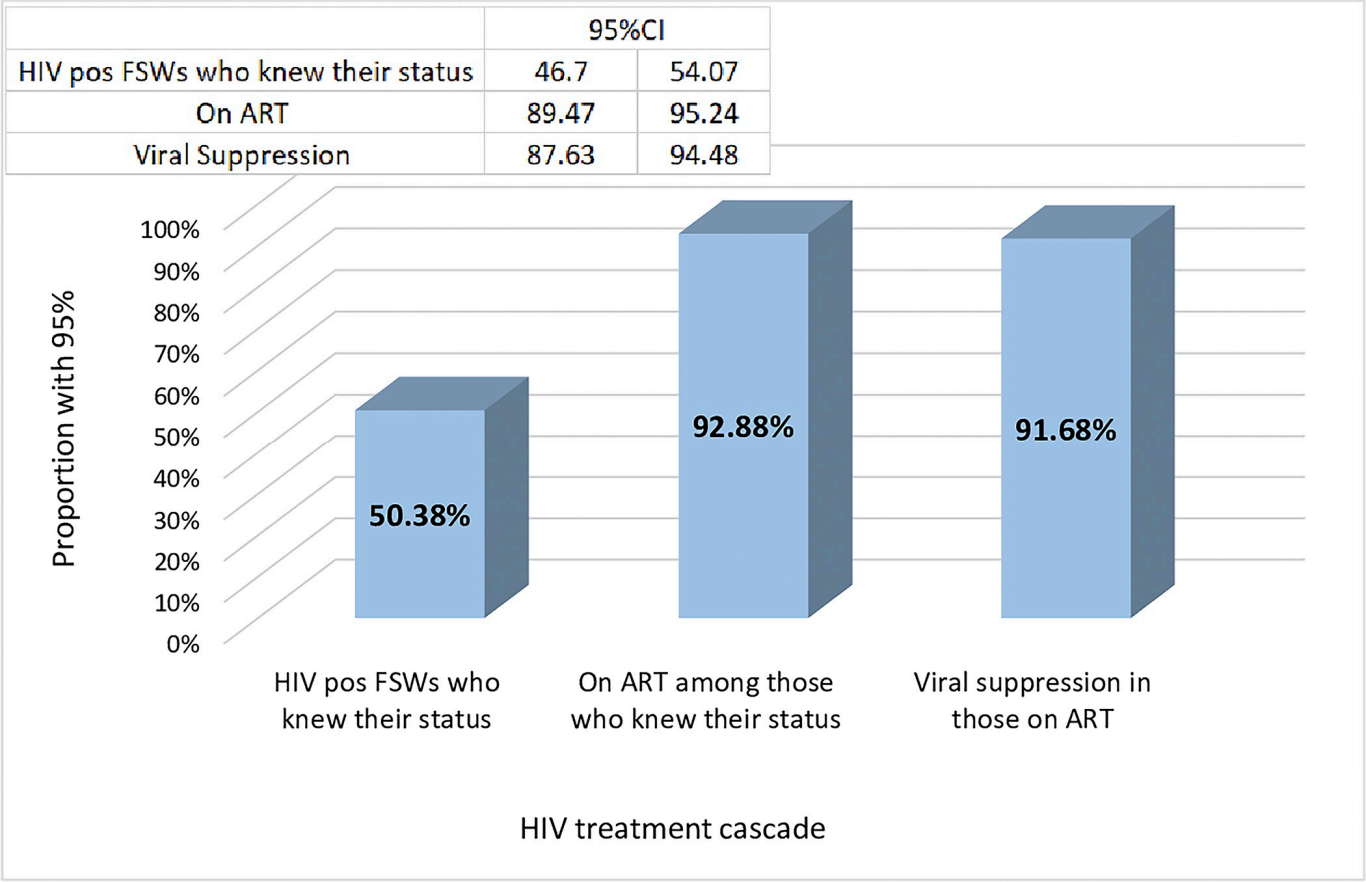
HIV treatment cascade against the 90-90-90 targets among female sex workers in Ethiopia, 2020.

Of the 575 (50.38%) FSWs who knew that they were HIV positive, 540 (92.88%) reported that they were on ART. Of all who were on ART, 503 (91.68%) had attained viral suppression of less than 1000 copies/mL (Fig 1).

**Fig 2:**
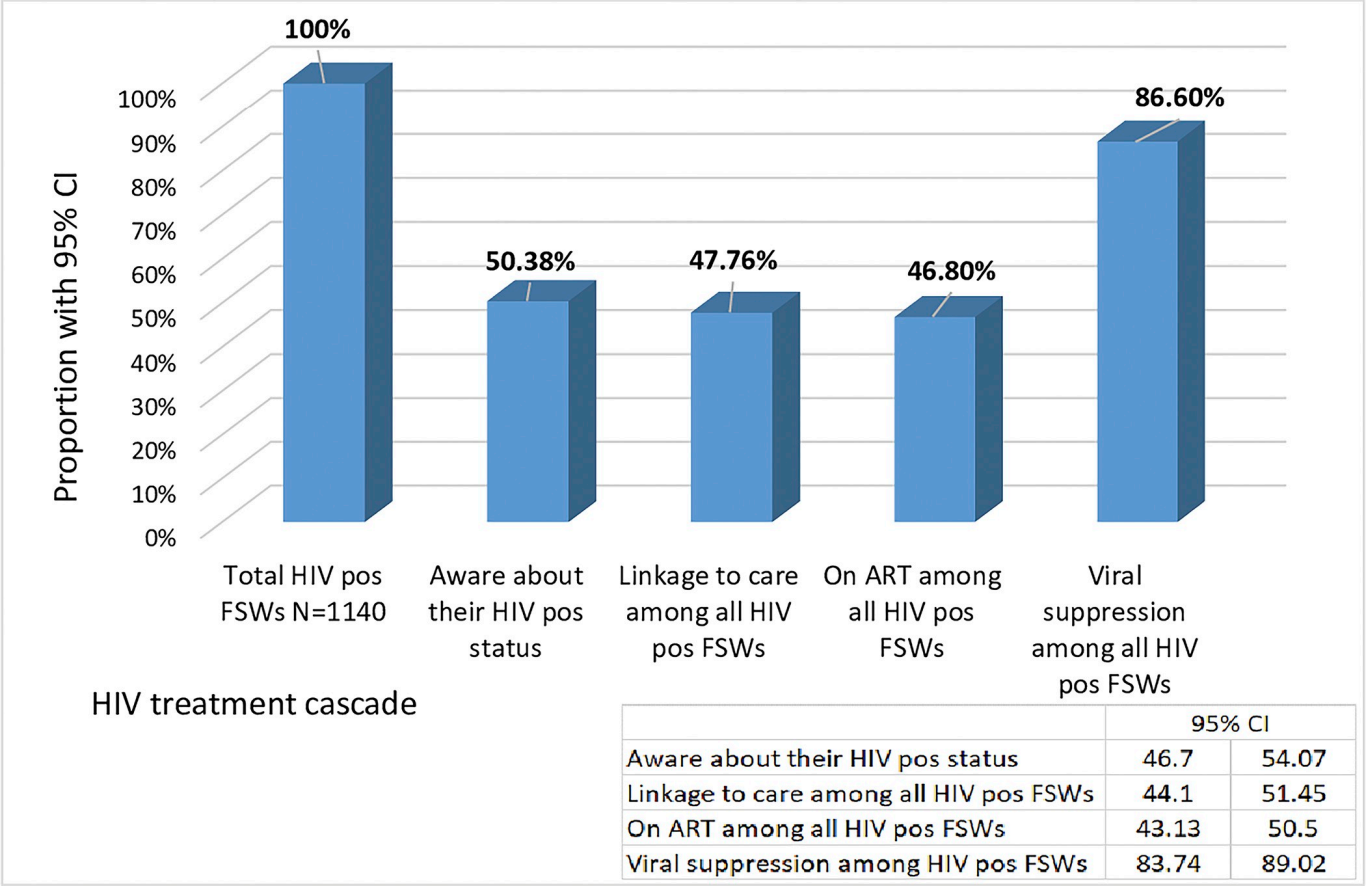
HIV treatment cascade among HIV-positive female sex workers in Ethiopia, 2020.

Even though linkage to care for treatment and viral suppression among FSWs who knew their HIV status was above 90%, only 47.76% and 46.80% of all FSWs living with HIV were linked to care and put on ART, respectively. Consequently, 86.6% of HIV-positive FSWs attained viral suppression, leaving 13.40% of them with an unsuppressed HIV viral load in their body (Fig 2).

### Factors associated with knowing HIV status

Age, marital status, HIV acquisition risk perception, and history of STIs were associated with HIV status awareness in the bivariate and multivariable analyses. The chi^2^ test output of the average monthly income of FSWs and HIV status awareness suggested the inclusion of the average monthly income in the multivariable logistic regression model. However, average monthly income did not maintain its significance in the multivariable analysis to be associated with HIV status awareness. Even though educational status was not associated with HIV status awareness in bivariate analysis, we kept it in the multivariable analysis model because this variable has a logical connection with access to HIV prevention.

Compared with FSWs who were younger than 25 years, the odds of knowing HIV status among FSWs above the age of 35 and between the ages of 25 and 35 were 3.20 (95% CI; 2.00, 5.13) and 1.65 (95% CI; 1.15, 2.36) times higher, respectively. The odds of knowing HIV status among widowed FSWs was 1.81 (95% CI; 1.05, 3.12) times the odds in those FSWs who never married. The odds of HIV-positive status awareness were also higher in FSWs who did not perceive the risk of HIV acquisition (AOR: 1.73, 95% CI; 1.28, 2.32) and who had a history of STIs (1.46, 95% CI; 1.05, 2.03) (Table 3).

**Table 3:** Factors associated with knowing HIV status among FSWs in Ethiopia, 2020 (N = 575).

Variable	Category	Knew HIV positive status		Crude Odds Ratio (95% CI)		Adjusted Odds Ratio (95% CI)	
		Yes	No		P-value		P-value
**Age**	Less than 25	94	154	1		1	
	25–35	331	322	1.68** (1.25, 2.27)	0.001	1.65** (1.15, 2.36)	0.007
	35 and above	150	89	2.76*** (1.91, 3.98)	< 0.001	3.20*** (2.00, 5.13)	<0.001
**MARITAL Status**	Never married	127	190	1		1	
	Married	26	18	2.16* (1.14, 4.10)	0.019	1.69 (0.81, 3.51)	0.163
	Separated	38	45	1.26 (0.78, 2.06)	0.347	0.84 (0.45, 1.55)	0.572
	Divorced	307	264	1.74*** (1.32, 2.30)	<0.001	1.28 (0.91, 1.81)	0.151
	Widowed	77	48	2.4*** (1.57, 3.67)	<0.001	1.81* (1.05, 3.12)	0.032
**Educational status**	Above high school, ≥9	96	91	1		1	
	Not read and write	323	303	0.86 (0.60, 1.24)	0.428	0.94 (0.61, 1.45)	0.796
	Primary school, 1–8	156	171	1.01 (0.73, 1.40)	0.950	1.31 (0.89, 1.94)	0.167
**Monthly income**	3000 and above	271	286	1		1	
	Less than 3000	304	279	1.15 (0.91, 1.45)	0.239	1.09 (0.81, 1.46)	0.579
**Risk perception**	Yes	325	291	1		1	
	No	250	127	1.76*** (1.35, 2.30)	<0.001	1.73*** (1.28, 2.32)	<0.001
**History of STIs**	No	438	463	1		1	
	Yes	137	102	1.42* (1.06, 1.89)	0.017	1.47* (1.04, 2.07)	0.029

*P<0.05

**P<0.01

***P<0.001

## Discussions

During the study period, only 50.38% of FSWs living with HIV were aware of their HIV-positive status. Treatment initiation and viral suppression rates among FSWs exceeded 90%, and the likelihood of knowing their HIV-positive status was higher among those who were older, widowed, perceived no risk of acquiring the virus, and had a history of sexually transmitted infections (STIs).

Regardless of possible barriers that could deter FSWs from testing for HIV, 89.59% of them had previously tested for HIV. It implies that a large proportion of FSWs are aware of the availability of HIV testing services and have had the opportunity to be tested. This finding is comparable to studies conducted in Kenya (88.6%) [26] and Zimbabwe (91.1%) [11]. However, only 50.38% of all HIV-positive FSWs knew they were HIV-positive prior to the study period, significantly diverging from the UNAIDS’ 90% test target [7]. In addition, the proportion of HIV-positive status awareness among FSWs living with the virus in this study is lower than the proportions in many African countries, including Benin (60.6%) [27], Zimbabwe (64%) [11], and South Africa (82%) [28].

The low level of knowledge of HIV status among HIV-positive FSWs could be explained by individual, societal, and structural barriers [29, 30]. In this study, among many individual attributes, older age, being widowed, having a lower perception of being at risk of acquiring HIV, and having a history of STIs were associated with knowing HIV status in HIV-positive FSWs. Similarly, a systematic review summarized a higher uptake of HIV testing among older FSWs [16]. The increased frequency of health facility visits among older FSWs may have contributed to a rise in HIV test uptake in older FSWs [31, 32], as healthcare providers are expected to offer the test as a standard service for high-risk populations [33]. Additionally, older FSWs have likely been exposed to intensive HIV prevention campaigns and testing advocacies [34], allowing them further opportunities to access this service. However, financial constraints in recent years have created a significant gap in HIV prevention and control services, including community mobilization activities that could potentially increase the uptake of HIV tests [35].

Similar to a multi-country study [36], being widowed in our study was associated with a higher awareness of HIV-positive status in FSWs. The existing research data on this issue supports multiple theories that may explain why widowed FSWs have higher awareness rates of HIV-positive status. The first theory is that widowed FSWs are more likely to accompany their sick partners to healthcare facilities, increasing their access to HIV tests [37, 38]. Another theory is that healthcare providers also use strategies like assisted partner notification and screening for FSWs to promote testing and make it more accessible among such key populations [39].

Increased awareness was also observed in FSWs with a previous history of STIs, which could be due to the counseling services offered by the health care providers, who treated them for the STIs, to take HIV tests. HIV testing offer is part of STI management in Ethiopia [40]. HIV and other STIs have similar facilitators and barriers to visiting health care and testing services [41]. This implies that FSWs with other STIs have more reasons to visit a health facility to test for HIV than FSWs without STIs. Therefore, FSWs who may not be able to visit health facilities due to illnesses should be given special attention during the planning and implementation of HIV prevention activities that aim to increase HIV test uptake.

FSWs’ perception of being at risk for HIV acquisition was associated with either an increase or decrease in HIV test uptake/HIV status awareness in different studies [42, 43]. A review reported that FSWs do not consider HIV testing if they believe that there is no or low risk of acquiring HIV in their social network [16]. Another review showed that FSWs’ perception of being at higher risk for HIV was associated with HIV testing and counseling service-seeking behavior and increased awareness due to a better understanding of the risk and prevention of HIV [42]. In societies with high stigma and discrimination, feeling at risk of acquiring HIV was associated with less awareness of HIV status. FSWs fear community intolerance that may come, if others know their HIV positive status. They are also insecure that they may lose their sexual partners if their positive status is known by others [42, 43]. Our study’s finding is similar to the latter scenario in that a lower perception of HIV acquisition was associated with a higher awareness of HIV-positive status. This implies that those FSWs who perceive that they are at higher risk of acquiring HIV are afraid to take an HIV test, possibly due to anticipated stigma. A study conducted in Addis Ababa, the capital city of Ethiopia, highlighted some of the barriers that affected the HIV test uptake of FSWs in the city. The barriers emphasized in the study were stigma from healthcare workers toward FSWs and the requisition of the national identification (ID) card to provide HIV care for HIV positives. At the same time, many FSWs do not possess it [44]. Activities that are relevant to destigmatizing HIV in the communities should be performed [16], along with other prevention activities to increase HIV test uptake. The requisition of ID before initiation of ART should be exempted, or alternative options for FSWs who do not have ID should be designed to increase their access to the services.

The majority of the FSWs who were aware of their HIV-positive status were put on ART (92.88%) and achieved viral suppression of less than 1000 copies per mL (91.68%). Compared to the UNAIDS treatment cascade targets (90-90-90) to end HIV by 2030 [7], our study showed that more than 90% of FSWs who knew their HIV-positive status were on ART and virally suppressed. This proportion is higher than the proportions reported by many other studies. To mention some: A study conducted in cross-border areas of 14 East African countries showed that 80.6% of FSWs who knew their status were on ART, and 84.8% of them were virally suppressed [9]; in Benin, 90.5% of FSWs who knew their status were on ART, and 81.8% of them had a suppressed viral load [27]; in Zimbabwe, 67.7% of FSWs who were aware of their status were on ART, and 77.8% of them had a suppressed HIV viral level [11].

This study is a national survey with adequate sample size. We have used the best sampling technique to study hidden populations to ensure the representation and generalizability of the study findings. The incentive was used to encourage the participation of reluctant members of subgroups, and a limited number of coupons was used to control oversampling due to volunteerism. A maximum of 16 waves were attained to penetrate the FSWs’ social network deeper. Seeds were selected from all known categories of FSWs and geographic locations in the city while convergence and equilibrium status for key variables (HIV status and consistent condom use) were attained, indicating that the sample population was independent of the original seeds. However, this study’s limitations include that some variables, like awareness of prior HIV status, were obtained through self-reporting by FSWs. Although it is unlikely that there will be a significant deviation from what is reported by them, it is possible that FSWs may have been reluctant to disclose their knowledge of HIV-positive status or that they were receiving ART due to the social stigma attached to the disease. For instance, given the low HIV positive status awareness (50.38%) and access to ART (46.80%), viral suppression of 86.6% is high, and this may indicate the existence of unreported HIV positive self awareness and being on ART. In addition, we did not collect exhaustive information on some variables that are thought to be essential to determining access to HIV tests, particularly those related to structural factors.

In conclusion, only 50.38% of HIV-positive FSWs knew their HIV status. However, more than 90% of them were linked to care and put on ART. The viral suppression among them was also high. HIV-positive status awareness was higher in older FSWs, widows, those who perceived having no risk of acquiring HIV, and those with a history of STIs. To improve the low proportion of HIV status awareness in HIV-positive FSWs, targeted interventions should be designed to reach the groups left behind, including younger FSWs, those who perceive having the risk of HIV acquisition, and those who have no history of STIs. Also, to reduce estimation bias due to not reporting true HIV status and access to ART, it is important to add anti-retroviral drug metabolites tests when assessing HIV treatment cascade.

## Supporting information

S1 Data (XLS)
